# Echocardiographic characteristics of patients with SARS-CoV-2 infection

**DOI:** 10.1007/s00392-020-01727-5

**Published:** 2020-08-14

**Authors:** Stephan Stöbe, Sarah Richter, Markus Seige, Sebastian Stehr, Ulrich Laufs, Andreas Hagendorff

**Affiliations:** 1 Department of Cardiology, Leipzig University Hospital, Liebigstr. 20, 04103 Leipzig, Germany; 2 Department of Internal Medicine I, Martha-Maria Hospital Halle-Dölau, Röntgenstr. 1, 06129 Halle (Saale), Germany; 3 Department of Anesthesiology and Intensive Care, Leipzig University Hospital, Liebigstr. 20, 04103 Leipzig, Germany

**Keywords:** SARS-CoV-2, COVID-19, Myocarditis, Deformation imaging, Myocardial strain, Rotation

## Abstract

**Background:**

Myocardial involvement induced by SARS-CoV-2 infection might be important for long-term prognosis. The aim of this observational study was to characterize the myocardial effects during SARS-CoV-2 infections by echocardiography.

**Results and methods:**

An extended echocardiographic image acquisition protocol was performed in 18 patients with SARS-CoV-2 infection assessing LV longitudinal, radial, and circumferential deformation including rotation, twist, and untwisting. Furthermore, LV deformation was analyzed in an age-matched control group of healthy individuals (*n* = 20). The most prevalent finding was a reduced longitudinal strain observed predominantly in more than one basal LV segment (*n* = 10/14 patients, 71%). This pattern reminded of a “reverse tako-tsubo” morphology that is not typical for other viral myocarditis. Additional findings included a biphasic pattern with maximum post-systolic or negative regional radial strain predominantly basal (*n* = 5/14 patients, 36%); the absence or dispersion of basal LV rotation (*n* = 6/14 patients, 43%); a reduced or positive regional circumferential strain in more than one segment (*n* = 7/14 patients, 50%); a net rotation showing late post-systolic twist or biphasic pattern (*n* = 8/14 patients, 57%); a net rotation showing polyphasic pattern and/or higher maximum net values during diastole (*n* = 8/14 patients, 57%).

**Conclusion:**

Myocardial involvement due to SARS-CoV-2-infection was highly prevalent in the present cohort—even in patients with mild symptoms. It appears to be characterized by specific speckle tracking deformation abnormalities in the basal LV segments. These data set the stage to prospectively test whether these parameters are helpful for risk stratification and for the long-term follow-up of these patients.

## Introduction

Coronavirus disease 2019 (COVID-19) is a systemic viral infection caused by SARS-CoV-2 (severe acute respiratory syndrome-coronavirus-2) which may lead to life-threatening severe acute respiratory syndromes, especially in high-risk patients [1–5]. COVID-19 patients showed myocardial involvement which is described as special type of acute myocarditis (AM) [6–10]. Myocardial damage can be caused directly by SARS-CoV-2 virus or by immunopathological sequelae of myocardial inflammation [8, 11, 12]. Myocardial involvement documented by increased levels of troponin T and brain-type-natriuretic peptide (NT-pro-BNP) or reduced left-ventricular (LV) ejection fraction (EF) is associated with adverse outcomes and increased mortality [13–16]. Therefore, it seems to be important to focus on the detection of myocardial damage at early stage of SARS-CoV-2-infection.

In addition, residual myocardial damage after recovery of the acute phase of the disease might have a significant impact on the patients’ prognosis probably due to SARS-CoV-2 induced myocardial fibrosis.

Currently, cardiac magnetic resonance (CMR) imaging represents the gold standard for the detection of AM [17, 18]. However, the use of CMR is limited by its availability, the risks of viral spread, and the state of the severely ill patients. Thus, imaging modalities like echocardiography might be favored under these conditions, because the devices are easier to clean and more suitable for repetitive investigations, especially if these patients need to be monitored at intensive-care units or in emergency settings.

The present paper describes the experience at the Leipzig University Hospital in detecting myocardial involvement in SARS-CoV-2-infected patients by echocardiography using a specialized extended imaging and analysis protocol to analyze different components of myocardial deformation [19]. The authors hypothesize that myocardial involvement might be detected by echocardiography due to specific abnormalities of regional LV function in SARS-CoV-2-infected patients.

## Methods

During the acute stage of the pandemic in April 2020, transthoracic echocardiography (TTE) was performed in 18 patients with SARS-CoV-2 infection at the Leipzig University Hospital and the Community Hospital Halle (Saale). Symptoms of SARS-CoV-2 patients varied from mild/moderate to severe respiratory symptoms requiring long-term mechanical ventilation. Thus, subgroup analyses were performed in patients with mild/moderate symptoms (no mechanical ventilation, *n* = 4) and severe symptoms (mechanical ventilation needed, *n* = 14).

All SARS-CoV-2-infected patients have been treated at isolation wards of different institutions, in which TTE was not routinely performed. After technical allocation of necessary ultrasound equipment, four expert cardiologists have performed bed-side 2D TTE with respect to the analysis of LV deformation by 2D speckle tracking by post-processing. The analysis of LV deformation was performed by two investigators experienced in speckle tracking. LV deformation could not have been analyzed by 3D echocardiography, because bed-side 3D TTE could not be performed at the isolation wards. For the detection of residual myocardial involvement beyond the acute-stage 3D TTE has only been performed in selected patients. Pathological echocardiographic findings were exemplarily confirmed by singular CMR results. Repetitive TTE was performed in three patients. Patient characteristics were collected from medical records, laboratory findings, ECG, and X-ray documentations (Table 1).

Furthermore, LV deformation was analyzed in an age-matched control group of healthy individuals (*n* = 20).

All subjects provided informed consent after full explanation of the purpose and order of all procedures. The study complies with the Declaration of Helsinki and the study design was approved by the locally appointed ethics committee (359/18-ek).

### Basic echocardiographic examination

TTE was performed using a Vivid S70 or Vivid q ultrasound system with an M5 or M5-S phased array probe (GE Healthcare Vingmed Ultrasound AS, Horten, Norway). Echocardiographic analyses were performed with the EchoPac software (Version 202, GE Healthcare Vingmed Ultrasound AS, Horten, Norway) using the quantitative analysis software package. LV dimensions (M mode and/or 2D measurements), LV volumes and LVEF (by biplane LV planimetry by the modified Simpson´s rule), relative wall thickness (RWT), LV mass (LVM) (by the Devereux formula), and LV mass index (LVMI) as well as LV remodeling index (LVRI) were assessed [20]. Diastolic function was characterized by maximum velocities of *E* and *A* waves (*V*_max_*E*, *V*_max_*A*), *E*/*A* ratio, *E*/*E*′ ratio (*V*_max_*E* and maximum myocardial velocities (*E*′) of the basal mitral annulus), isovolumetric relaxation time (IVRT) obtained by pulsed-wave (PW) tissue Doppler imaging, and left atrial (LA) volumes determined by biplane LA planimetry [21]. Right-ventricular (RV) function was assessed by tricuspid annular plane systolic excursion (TAPSE), pulmonary acceleration time (AccT), and by estimation of systolic pulmonary artery pressure (sPAP) assessed by maximum velocity of tricuspid regurgitation using continuous-wave (CW) Doppler.

### Myocardial deformation analysis

Global (GLS) and regional (rLS) longitudinal deformation was assessed by determination of layer strain in all apical views. Global (GCS) and regional (rCS) circumferential layer strain and global (GRS) and regional (rRS) radial strain were determined in apical, mid, and basal parasternal short-axis views. For analysis of rotation and rotation rate (twist and untwisting), only apical short -axis views were accepted, which definitively were within the apical third of the LV. Basal short-axis views required documentation of the LV wall during the complete cardiac cycle. In addition, RV GLS was assessed in apical four-chamber views (4-chV). The endocardial contour was manually adjusted, whereas only segments with accurate tracking by carefully visual evaluation were accepted to exclude imaging artifacts. Tracking areas were adjusted to enable full myocardial tracking excluding epicardial as well as valvular or atrial structures. Complete deformation analysis could be performed in 14 patients—one patient was excluded due to left bundle branch block (LBBB) and three due to insufficient image quality. All excluded patients belonged to the patient group with severe symptoms (Table 2).

### Statistical analysis

All statistical analyses were performed using SPSS Statistics version 24.0 (IBM, Armonk, NY). Continuous variables were expressed as mean value ± standard deviation (SD) and were compared between groups using Student’s *t* test. Statistical significance was accepted for *p* value < 0.05. Intra-observer variability and inter-observer (by another investigator blinded to each other’s results) variability were assessed in five patients.

## Results

Characteristics of all SARS-CoV-2-infected patients are presented in Table 1. Troponin T, NT-pro-BNP, C-reactive protein (CrP), procalcitonin (PCT), interleukin-6 (IL-6), and d-dimer were significantly increased in patients with severe in comparison to those with mild/moderate symptoms (Table 1). Cardiac dimensions and function assessed by conventional TTE were within normal ranges except LVRI (Table 1). In patients with severe symptoms, *E*/*E*′ was significantly higher in comparison to those with mild/moderate symptoms. RV echocardiographic parameters were not significantly different between both groups (Table 1). However, GLS of the free RV wall was mildly reduced (RV GLS: between − 17 and − 23%) in four patients with severe symptoms needing mechanical ventilation. All of these four patients showed elevated Troponin T- and NT-pro-BNP values. In all the other patients with severe symptoms, normal mean RV GLS values of at least − 28% were observed. In addition, two patients with mild symptoms also showed mildly reduced RV GLS (between − 22 and − 23%).

**Table 1:** Patient characteristics, symptoms, laboratory findings, comorbidities, as well as conventional 2D echocardiographic and modern deformation parameters of SARS-CoV-2-patients are shown

	COVID-19 (*n* = 18)	COVID-19: severe (*n* = 14)	COVID-19: mild (*n* = 4)	*p* value
Male	14 (78%)	11 (79%)	1 (25%)	0.90
Age (years)	64 ± 19.1	71 ± 15.2	41 ± 11.8	< 0.05
Temperature (°C)	38.1 ± 1.1	38.3 ± 1.1	36.9 ± 0.3	< 0.05
Oxygen saturation (%)	93 ± 6.7	91 ± 6.6	99 ± 0.8	< 0.05
Respiratory rate (min^−1^)	21 ± 6.2	24 ± 5.1	15 ± 3.3	< 0.05
Blood pressure systolic/diastolic (mmHg)	136/72 ± 18.7/10.0	134/69 ± 21.0/7.6	142/82 ± 10.3/11.5	0.44/0.18
Heart rate (bpm)	89 ± 14.8	90 ± 15.7	84 ± 11.5	0.47
Dyspnoea	13 (72%)	7 (100%)	6 (55%)	0.41
Cough	10 (56%)	5 (71%)	5 (45%)	0.23
Fever	10 (56%)	4 (36%)	6 (55%)	0.83
Fatigue	5 (28%)	3 (43%)	2 (18%)	0.90
Angina pectoris	0 (0%)	0 (0%)	0 (%)	–
Leucocytes (exp9/l)	8.6 ± 4.3	8.9 ± 4.5	7.3 ± 3.1	0.52
C-reactive protein (mg/l)	107.4 ± 96.9	131.8 ± 91.1	1.7 ± 0.8	< 0.05
Procalcitonin (ng/ml)	4.9 ± 15.4	5.3 ± 15.9	< 0.05	< 0.05
Interleukin-6 (pg/ml)	127.8 ± 122.1	127.8 ± 122.3	< 7.0	< 0.05
Creatine kinase (µkat/l)	3.8 ± 6.6	4.9 ± 7.6	1.1 ± 0.3	0.33
Troponin T (pg/ml)	36 ± 23	36 ± 23	< 3	< 0.05
NT-proBNP (pg/ml)	1724 ± 2058	1724 ± 2118	< 50	< 0.05
d-Dimer (mg/l)	4.5 ± 5.0	5.2 ± 5.2	0.3 ± 0.03	< 0.05
Arterial hypertension	13 (72%)	12 (86%)	1 (25%)	0.09
Paroxysmal atrial fibrillation	4 (22%)	4 (29%)	0 (0%)	< 0.05
Valvular heart disease (> moderate)	0 (0%)	0 (0%)	0 (0%)	–
Coronary artery disease	2 (11%)	2 (14%)	0 (0%)	0.17
Myocardial infarction	0 (0%)	0 (0%)	0 (0%)	–
Peripheral artery disease	3 (17%)	3 (21%)	0 (0%)	0.17
Dyslipidemia	4 (22%)	3 (21%)	1 (25%)	0.90
Diabetes mellitus	5 (28%)	4 (29%)	1 (25%)	0.48
Chronic kidney disease < G2 (according to KDIGO)	7 (39%)	7 (50%)	0 (0%)	< 0.05
Chronic obstructive pulmonary disease	1 (5%)	1 (7%)	0 (0%)	0.39
Stroke	3 (17%)	3 (21%)	0 (0%)	0.09
Pericardial effusion	1 (5%)	1 (7%)	0 (0%)	0.39
Pleural effusion	2 (11%)	2 (14%)	0 (0%)	0.19
Max. left atrial volume index (ml/m^2^)	22 ± 9.4	24 ± 9.7	15 ± 4.2	< 0.05
Relative wall thickness (RWT)	0.45 ± 0.04	0.46 ± 0.02	0.43 ± 0.07	0.46
Left-ventricular mass index (g/m^2^)	97 ± 19.0	103 ± 16.8	76 ± 6.3	< 0.05
Left-ventricular remodeling index (g/ml)	2.2 ± 0.7	2.3 ± 0.7	1.7 ± 0.3	< 0.05
Indexed left-ventricular end-diastolic volume (ml/m^2^)	47 ± 11.5	47 ± 12.2	46 ± 10.3	0.69
Left-ventricular ejection fraction (%)	62 ± 6.5	63 ± 6.7	58 ± 4.9	0.16
*E*/*A* ratio	0.95 ± 0.3	0.88 ± 0.2	1.15 ± 0.3	0.15
Left-ventricular end-diastolic pressure *E*/*e*′	8.6 ± 2.6	9.2 ± 2.6	6.7 ± 1.6	< 0.05
Isovolumetric relaxation time (s)	69 ± 19.4	65 ± 18.6	82 ± 18.4	0.17
Mitral annular plain systolic excursion (mm)	12 ± 2.2	12 ± 2.3	13 ± 1.7	0.72
Tricuspid annular plain systolic excursion (mm)	22 ± 3.2	22 ± 3.5	22 ± 2.4	0.89
Pulmonary acceleration time (s)	111 ± 19.9	107 ± 19.9	124 ± 14.9	0.11
Systolic pulmonary artery pressure (mmHg)	26 ± 8.7	26 ± 9.2	26 ± 7.8	0.95

	COVID-19 (*n* = 14)	COVID-19: severe (*n* = 10)	COVID-19: mild (*n* = 4)	*p* value
Global longitudinal strain (%)	− 19.7 ± 3.1	− 19.6 ± 3.1	− 20.0 ± 3.5	0.88
Global radial strain: apical (%)	43.0 ± 18.3	40.7 ± 20.3	50.7 ± 6.7	0.21
Global radial strain: basal (%)	35.7 ± 20.1	34.6 ± 22.0	41 ± 0.0	0.38
Global circumferential strain: apical (%)	− 18.5 ± 3.7	− 18.2 ± 3.7	− 19.3 ± 4.5	0.72
Global circumferential strain: basal [%]	− 10.8 ± 5.3	− 10.2 ± 5.7	− 13.5 ± 2.1	0.23
Rotation apical (°)	10.0 ± 8.6	11.9 ± 7.8	3.6 ± 9.7	0.28
Rotation basal (°)	− 5.4 ± 6.1	− 5.2 ± 6.5	− 6.5 ± 4.9	0.78
Right-ventricular GLS (%)	− 26.9 ± 5.8	− 26.6 ± 5.9	− 27.5 ± 6.1	0.76

Furthermore, patients were divided into two subgroups with respect to pulmonary condition (severe—need of mechanical or non-invasive ventilation vs. mild/moderate—no need of mechanical or non-invasive ventilation)

*COVID-19* coronavirus disease, *GLS* global longitudinal strain

Mean values ± standard deviation and numerical (percentage) distribution are shown. Statistical significance was accepted for *p* value of < 0.05

**Fig. 1:**
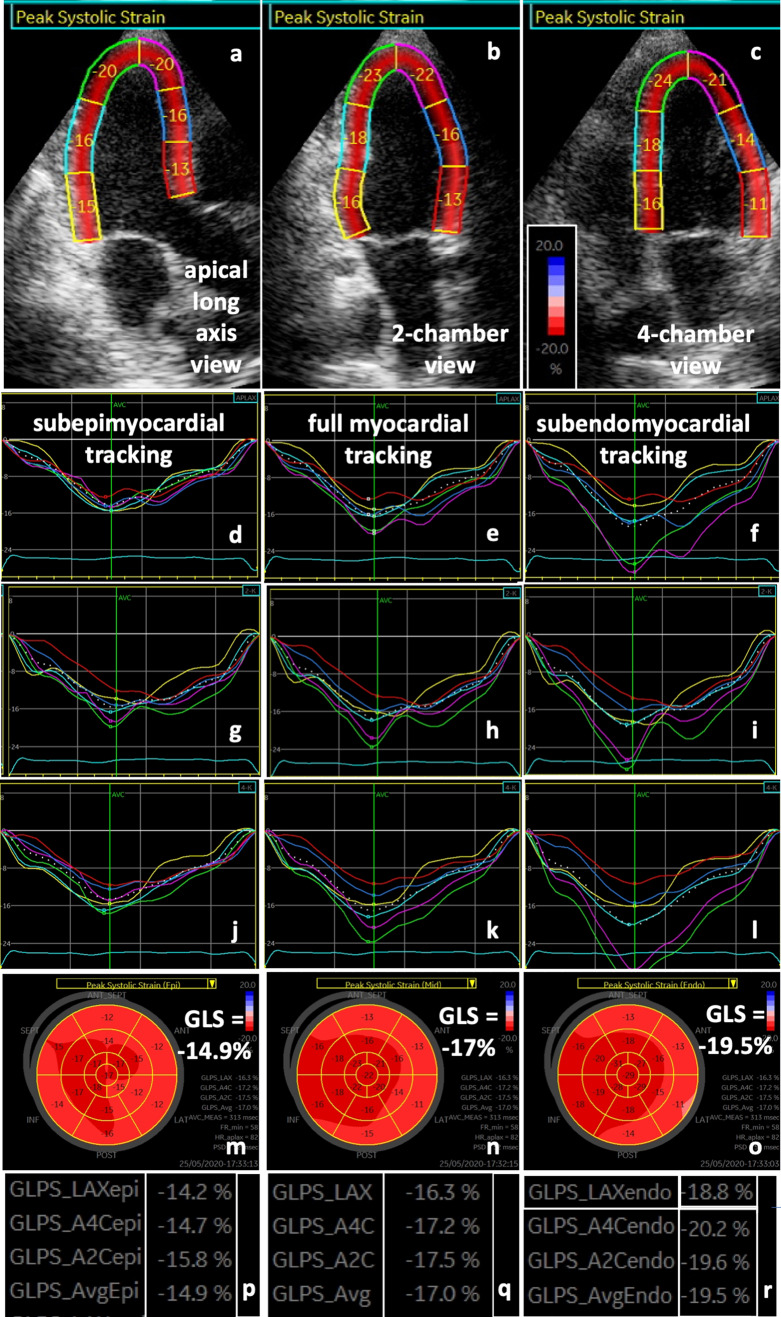
Longitudinal deformation pattern of a SARS-CoV-2-infected patient with severe symptoms: regional peak systolic longitudinal strain values are presented in the apical long axis (**a** aLAX), 2- (**b** 2ChV) and 4-chamber view (**c** 4ChV). Below regional left-ventricular (LV) longitudinal strain (rLS) curves of subepimyocardial (**d**, **g**, **j**), full myocardial (**e**, **h**, **k**) and subendomyocardial tracking (**f**, **i**, **l**) of the aLAX (**d**–**f**), 2- (**g**–**i**) and 4-ChV (**j**–**l**) show reduced basal rLS values. Subepimyocardial (*m*), full myocardial (*n*), and subendomyocardial (*o*) LS bull’s eye patterns and corresponding LS values (*p*–*r*) are shown with predominantly reduced rLS inferolateral, anterolateral, and anterior

**Fig. 2:**
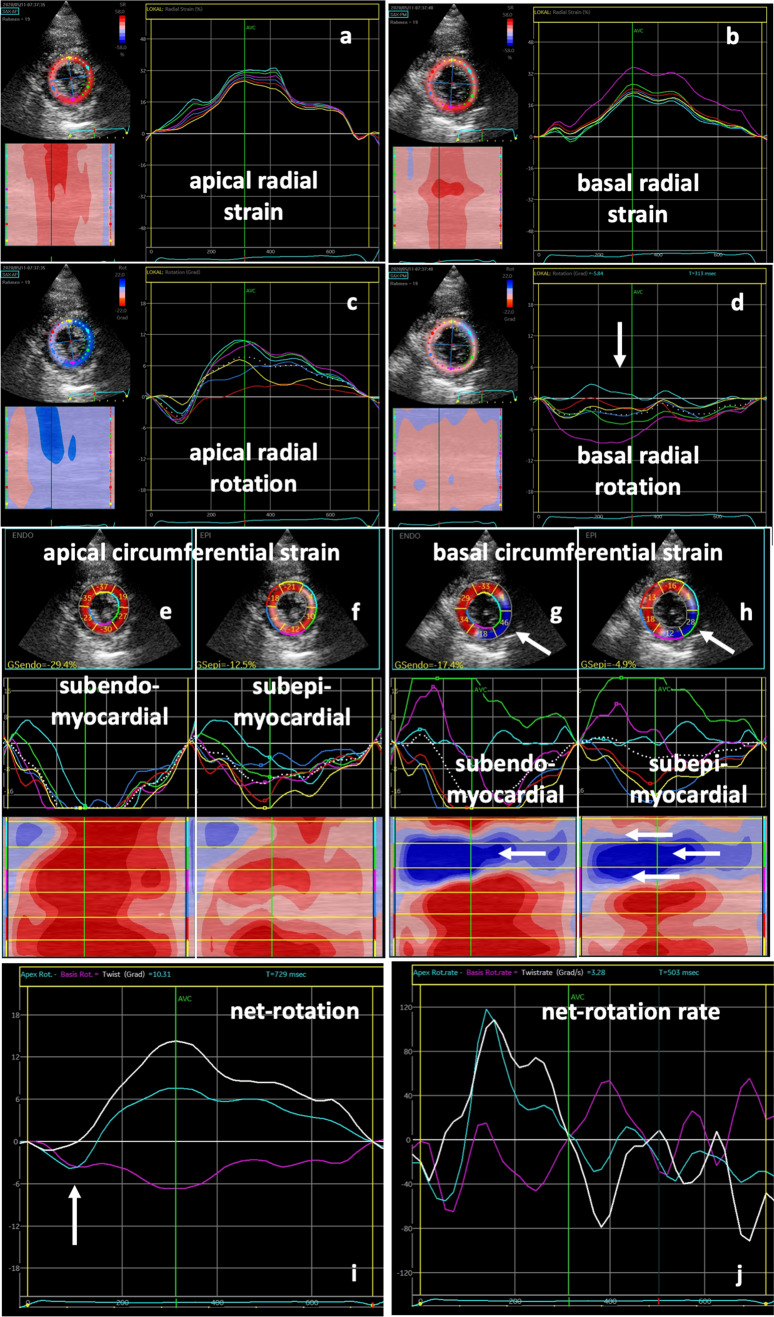
Rotational deformation pattern of the same SARS-CoV-2-infected patient with COVID-19 pulmonary disease as in Fig. 1: normal radial strain patterns are documented in apical (**a**) and basal (**b**) left-ventricular (LV) segments. Apical rotation was also normal (**c**), whereas basal rotation showed a regional dispersion with almost no rotation septal and anterior (**d**, white arrow). Below parasternal short-axis views with segmental subendomyocardial (**e**, **g**) and subepimyocardial (**f**, **h**) circumferential strain values, strain curves, and color-M-Modes are shown. Abnormal deformation is documented in basal anterior, anterolateral, and inferolateral (**g**, **h** white arrows). Line graphs of apical (blue) and basal rotation (magenta) as well as net rotation (twist) (white) (**i**) and corresponding line graphs of rotation rate (**j**) document abnormal twist (biphasic apical rotation, **i** white arrows) and normal untwisting (**j**)

In contrast to conventional echocardiography, deformation imaging (*n* = 14) revealed several interesting findings potentially documenting myocardial involvement in SARS-CoV-2-infected patients with mild/moderate and severe symptoms (Table 2; Figs. 1, 2):

**Table 2:** Analyses of myocardial deformation in SARS-CoV-2-infected patients

		rLS (C1)	LV GLS	rRS apical (C2)	GRS apical	Rotation apical	Mean rotation apical	rCS apical (C3)	GCS apical	rRS basal (C2)	GRS basal	Rotation basal (C4)	Mean rotation basal	rCS basal (C3)	GCS basal	Twist (C5)	Rotation rate (C6)	RV GLS
1	MechV	il, al (basal)	− 16	Norm	67	Norm	9	Path a	− 17	Path	14	Norm	− 8	Norm	− 14	Norm	Norm	− 29
2	MechV	il (basal)	− 19	Norm	38	Norm	15	Norm	− 22	Norm	62	Path	− 3	Path l	− 15	Norm	Norm	− 31
3^a^	MechV	a, al (basal)	− 17	Norm	31	Path	8	Path a	− 15	Norm	46	Path	2	Path a, al	0	Path	Path	− 18
4^b^	MechV	a, al (mid− basal)	− 23	–	–	–	–	–	–	–	–	–	–	–	–	–	–	− 17
5	NIV	a, as, is (basal)	− 19	Norm	37	Norm	13	Norm	− 23	Path	− 16	Path	− 4	Path i, il, al	− 11–	Path	Path	− 32
6^b^	NIV	i, al, a (basal)	− 15	–	–	–	–	–	–	–	–	–	–	–	–	–	–	–
7	O_2_	as (basal)	− 26	Path	18	Norm	13	Norm	− 16	Path	36	Norm	− 8	Path il	− 15	Norm	Norm	− 29
8	O_2_	as, al (basal)	− 20	Norm	73	Norm	13	Norm	− 21	Path	44	Path	6	Path i, il, al	− 13	Path	Path	− 30
9	O_2_	il, al (basal)	− 23	Norm	66	Norm	19	Norm	− 23	Norm	49	Norm	− 12	Path l	− 17	Path	Path	− 35
10^c^	O_2_	–	–	–	–	–	–	–	–	–	–	–	–	–	–	–	–	–
11	O_2_	aI (basal)	− 21	Norm	22	Path	− 7	Path a, as	− 13	Norm	36	Norm	− 13	Path il, al, a	− 8	Norm	Norm	− 21
12	O_2_	Is (basal)	− 19	Norm	30	Norm	14	Path is, as	− 15	Norm	28	Norm	− 12	Path i, il	− 4	Norm	Norm	− 23
13	O_2_	a, as, is (basal)	− 21	Norm	25	Norm	22	Norm	− 17	Norm	47	Path	0	Path il, al, a	− 5	Path	Path	− 28
14^b^	O_2_	–	–	–	–	–	–	–	–	–	–	–	–	–	–	–	–	–
15	–	il (basal)	− 18	Norm	45	Norm	12	Norm	− 15	Norm	41	Norm	− 10	Path a	− 12	Norm	Norm	− 22
16^a^	–	as, is (basal)	− 18	Norm	49	Path	− 7	Norm	− 24	Norm	41	Path	− 3	Path il	− 15	Path	Path	− 33
17	–	as, is (basal)	− 24	Norm	58	Path	6	Norm	− 19	Path	24	Norm	− 16	Path i, il	− 3	Path	Path	− 23
18	–	Norm	− 24	Norm	30	Path	2	Norm	− 19	Norm	31	Norm	− 13	Path il	− 16	Path	Path	− 33

Findings of regional longitudinal strain (rLS), left-ventricular global longitudinal strain (LV GLS), regional radial strain of apical left-ventricular (LV) segments (rRS apical), global radial strain of apical LV segments (GRS apical), apical rotation, mean apical rotation, regional circumferential strain of apical LV segments (rCS apical), global circumferential strain of apical LV segments (GCS apical), regional radial strain of basal left-ventricular (LV) segments (rRS basal), global radial strain of basal LV segments (GRS basal), basal rotation, mean basal rotation, regional circumferential strain of basal LV segments (rCS basal), global circumferential strain of basal LV segments (GCS basal), twist, rotation rate, and global longitudinal strain of the free right-ventricular wall (RV GLS)

LV segments are labeled by *a* anterior, *as* anteroseptal, *I* inferior, *il* inferolateral, *is* inferoseptal and *al* anterolateral. *MechV* mechanical ventilation, *NIV* non-invasive ventilation, *O*_*2*_ oxygen supply via mask, *path* pathological finding, *norm* normal finding, *C1* pathological criterium 1 described by qualitative alterations (see “Results”), *C2* pathological criterium 2, *C3* pathological criterium 3, *C4* pathological criterium 4, *C5* pathological criterium 5, *C6* pathological criterium 6

^a^Patients with CMR

^b^Patients with insufficient image quality

^c^Patient with left bundle branch block (LBBB)

1. rLS was reduced in more than one of the basal LV segments—predominantly within the subepimyocardium documented by layer-strain analysis (*n* = 10/14 patients, 71%).
2. rRS curves showed biphasic pattern with maximum post-systolic rRS (= early systolic LV wall thinning) or a negative systolic rRS (= complete systolic LV wall thinning) (*n* = 5/14 patients, 36%).
3. Absence or dispersion of basal rotation was observed (*n* = 6/14 patients, 43%).
4. rCS was severely reduced or was positive in more than one of LV segments—predominantly in the mid and basal LV segments (*n* = 7/14 patients, 50%).
5. The net rotation showed a late post-systolic twist or a biphasic pattern (*n* = 8/14 patients, 57%).
6. The net-rotation curve showed an undulating polyphasic pattern during systole and/or higher maximum net values during diastole than during systole (= “chaotic” undulating diastolic pattern) (*n* = 8/14 patients, 57%).

**Fig. 3:**
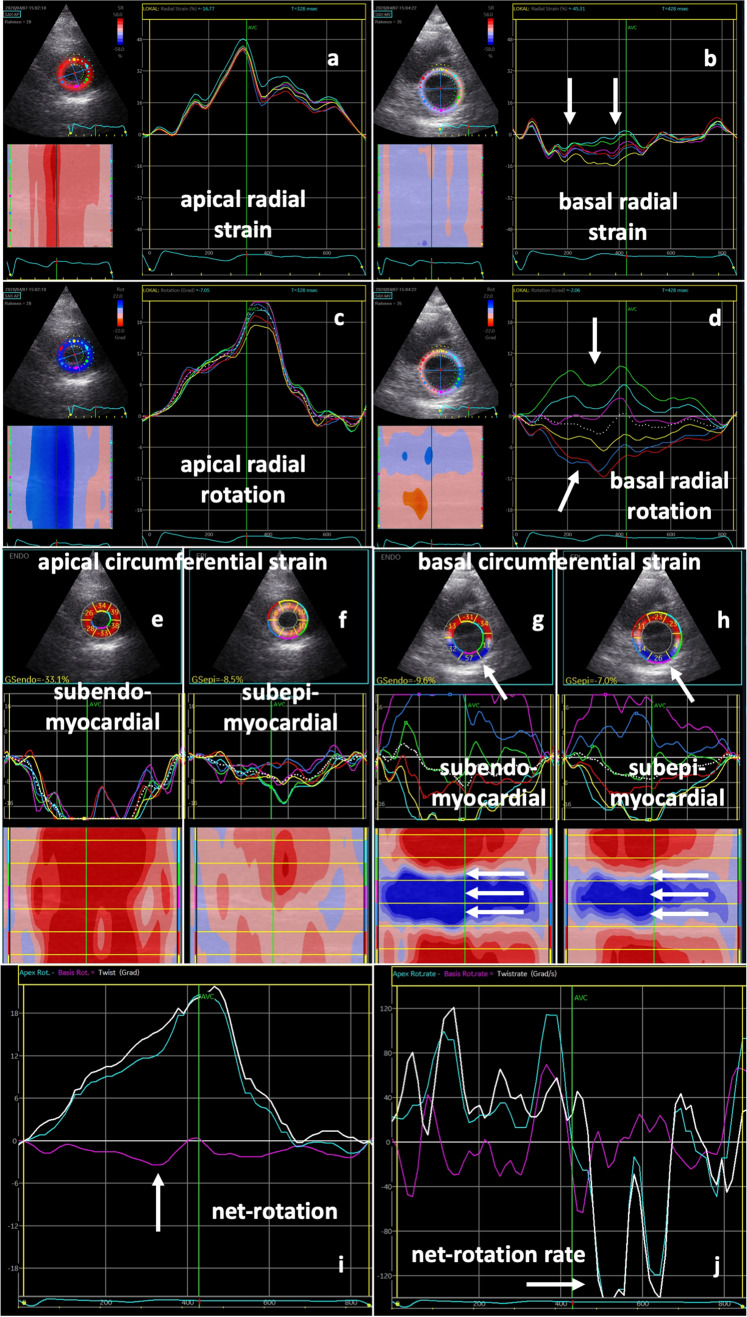
Rotational deformation pattern in another SARS-CoV-2-infected patient with severe symptoms: normal radial strain patterns are documented in apical (**a**), whereas abnormal patterns are shown in basal (**b**) left-ventricular (LV) segments. Apical rotation was also normal (**c**), whereas basal rotation showed a regional dispersion with inverse rotation in various LV segments (**d**, white arrows). Below parasternal short-axis views with segmental subendomyocardial (**e**, **g**) and subepimyocardial (**f**, **h** ) circumferential strain values, strain curves, and color-M-Modes are shown. Abnormal deformation is documented basal inferior, inferolateral, and anterolateral (**g**, **h** white arrows). Line graphs of apical (blue) and basal rotation (magenta) as well as net rotation (twist) (white) (**i**) and corresponding line graphs of rotation rate (**j**) document abnormal basal rotation with compensated twist by pronounced apical rotation (**i** white arrows) and a “chaotic” pattern of net-rotation rate during diastole (**j**)

The last two criteria can only be used, if rotation of the apical segments could have been correctly analyzed. Thus, the first four criteria can be classified as major criteria. Pathological deformation (> 2 major criteria) was observed in 13 of 14 (93%) SARS-CoV-2-infected patients and in all patients with severe symptoms (Fig. 3). Most of them presented comparable patterns of LV dysfunction similar to LV distribution of a “reverse basal tako-tsubo-like syndrome” (*n* = 7 with severe, *n* = 1 with mild/moderate symptoms).

**Fig. 4:**
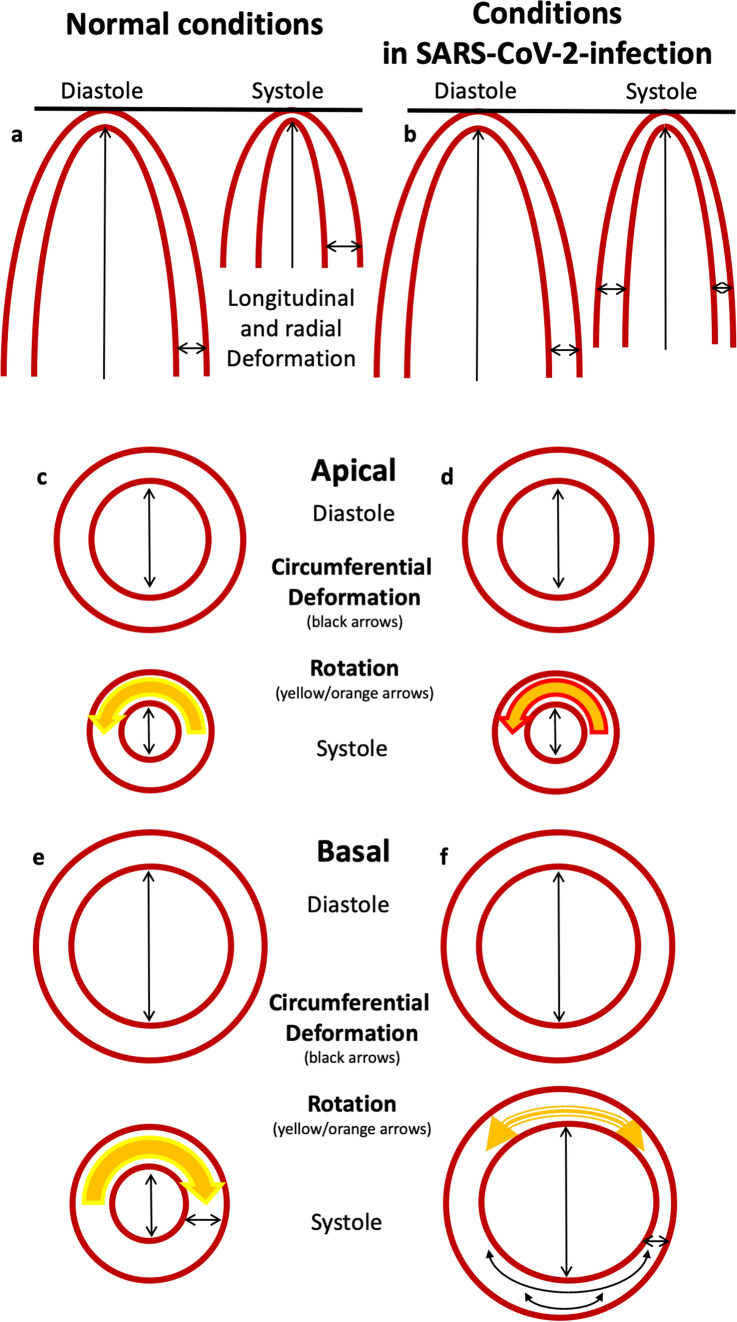
Scheme of longitudinal and radial (**a**, **b**) and apical (**c**, **d**) and basal (**e**, **f**) circumferential and rotational left-ventricular (LV) deformation under normal conditions (**a**, **c**, **e**) and in patients with SARS-CoV-2-infection (**b**, **d**, **f**). Normal changes of longitudinal strain (LS) are documented by longitudinal LV shortening (↑) and normal changes of radial strain (RS) by LV wall thickening (↔) (**a**). In patients with SARS-CoV-2-infection, regional LS and RS are reduced (**b**). In comparison to normal conditions (**e**), basal circumferential strain (CS—black arrows) and clockwise rotation (colored arrows) are severely reduced in patients with SARS-CoV-2 infection (**f**). In addition, especially basal rRS is reduced (↔) in patients with SARS-CoV-2 infection (**f**)

**Fig. 5:**
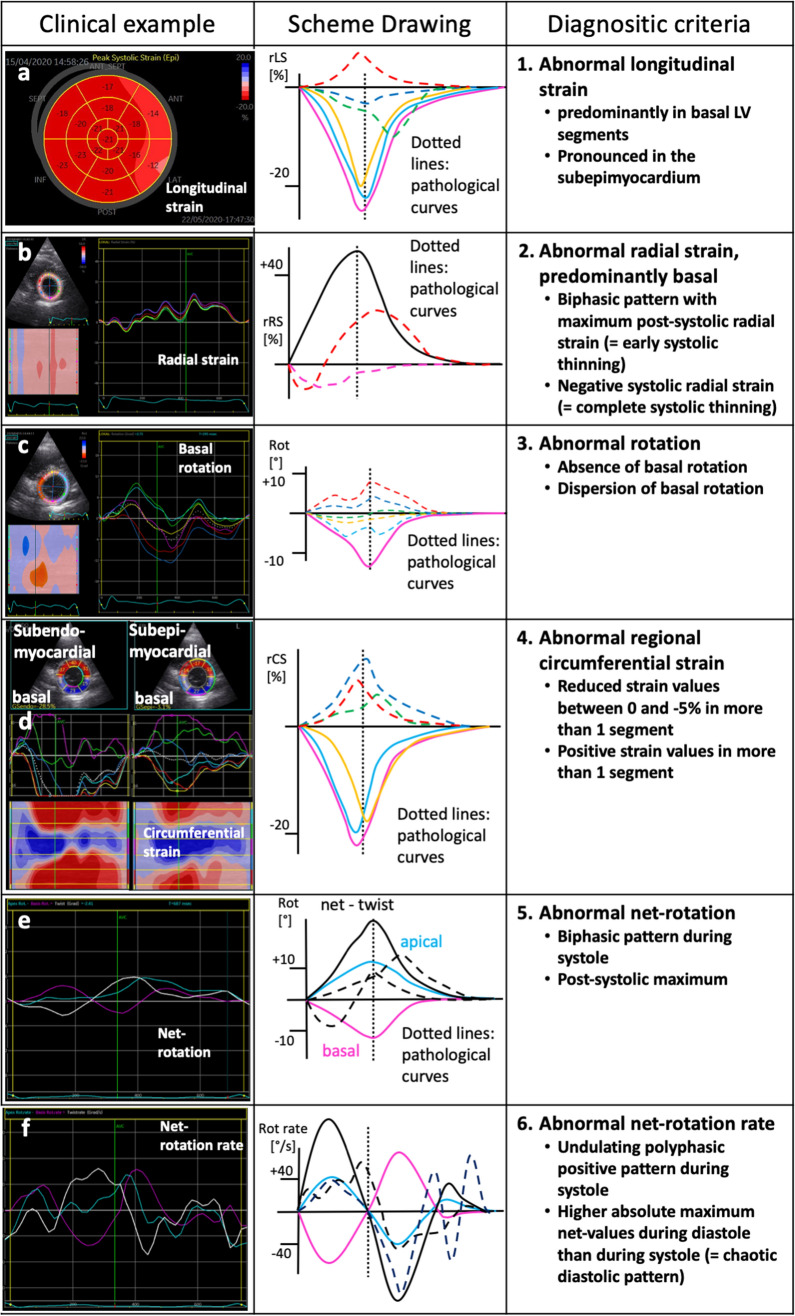
Overview of typical echocardiographic examples of abnormal deformation criteria in SARS-CoV-2-infected patients (left column) including representative schemes (mid column) and description (right column): reduced regional longitudinal strain predominantly in basal LV segments (**a**), reduced radial strain predominantly in basal LV segments (**b**), absence or dispersion of basal rotation (**c**), regional positive circumferential strain predominantly mid/basal lateral and anterior (**d**), biphasic net rotation (**e**), and polyphasic curves during systole, higher maximum, and “chaotic” curves of net-rotation rate during diastole (**f**)

In 5 of 14 (36%) COVID-19 patients, the qualitative pattern of rRS was abnormal showing no alteration of LV wall thickness or in some cases even wall thinning (Figs. 4, 5). The alterations of rRS were often associated with rotation abnormalities (Figs. 4, 5).

Abnormal rCS was most often observed in the inferior, and infero- and anterolateral basal LV segments showing lengthening of the respective LV segments during systole (*n* = 7/14 patients, 50%) (Figs. 4, 5). Abnormal rCS was usually observed in all layers. Abnormal rCS of the subepimyocardial layers was rarely observed (*n* = 3/14 patients, 21%). CS was abnormal in 90% of patients with severe symptoms (*n* = 9/10), mostly with transmural involvement.

Twist and untwisting patterns—documented by net rotation and net-rotation rate—were abnormal due to impaired rotation of basal LV segments (Figs. 4, 5). Surprisingly, qualitative abnormal deformation patterns were also found in 3 of 4 (75%) patients with only mild/moderate symptoms (Table 2).

**Fig. 6:**
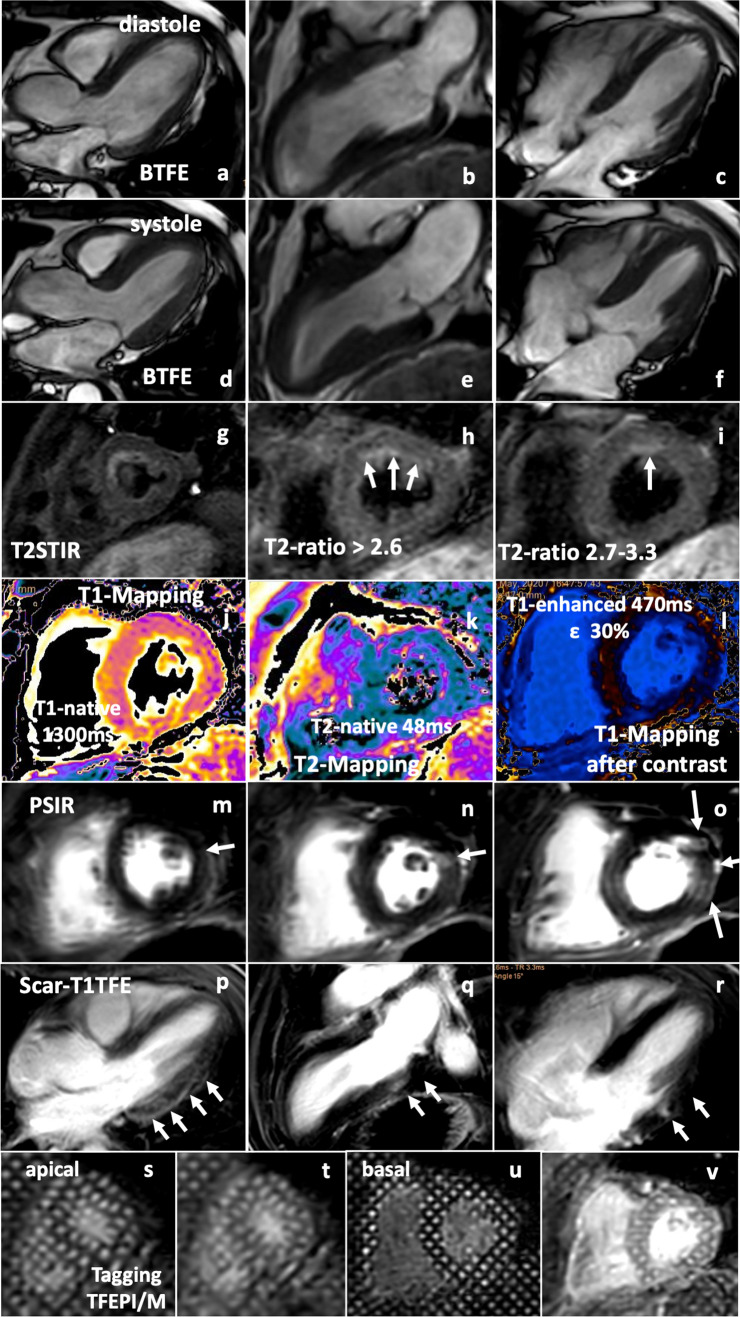
CMR findings of the same SARS-CoV-2-infected patient with COVID-19 pulmonary disease as in Fig. 1: BTFE images of long-axis view (LAX—**a**, **d**), two-chamber view (2-ChV—**b**, **e**) and four-chamber view (4ChV—**c**, **f**) during diastole (**a**–**c**) and systole (**d**–**f**). T2STIR sequences of apical (**g**), mid (**h**), and basal (**i**) short-axis views (SAX). Edema is pronounced mid/basal septal-anterior (white arrows). Representative images of T1- (**j**), T2-mapping (**k**), and T1-mapping after contrast (**l**); PSIR sequences of mid/basal SAX views (**m**–**o**) documenting regional patchy late enhancement predominantly lateral (white arrows). Scar-T1TFE images of LAX (**p**), 2-ChV (**q**), and 4-ChV (**r**) document late enhancement mid/basal inferolateral (**p**), inferior (**q**), and anterolateral (**r**). Tagging images (**s**–**v**) of apical (**s**, **t**) and basal SAX (**u**, **v**) document abnormal basal rotation

In two patients (one with severe and one with mild/moderate symptoms), echocardiographic findings could be confirmed by CMR (Fig. 6) illustrating regional edema and delayed enhancement predominantly in basal/mid LV segments as well as by impaired basal LV rotation in tagging sequences.

**Fig. 7:**
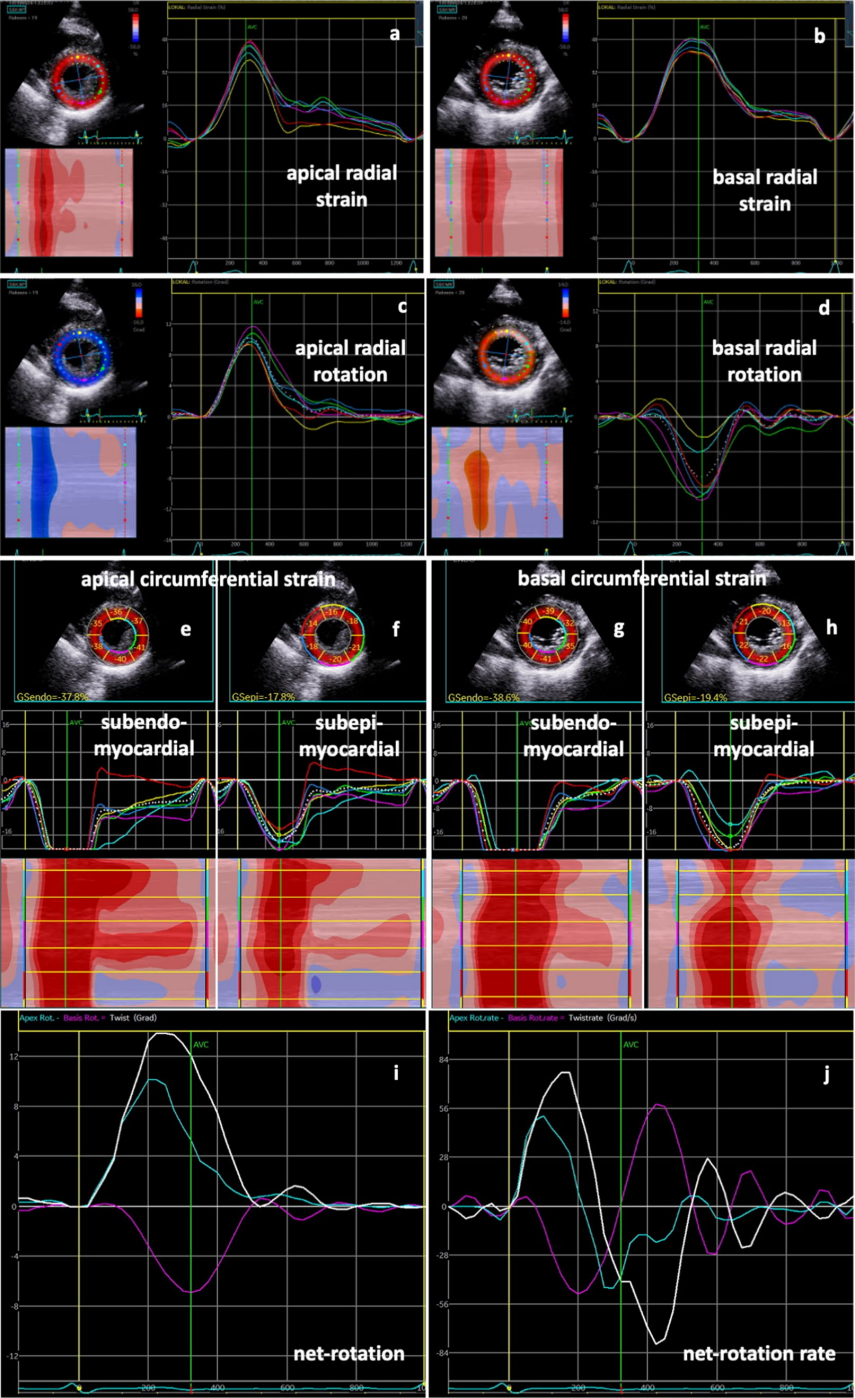
Rotational deformation pattern of a healthy individual: radial strain patterns in apical (**a**) and basal (**b**) left-ventricular (LV) segments as well as apical (**c**) and basal rotation (**d**) are documented. Below parasternal short-axis views with apical (**e**, **f**) and basal (**g**, **h**) segmental subendomyocardial (**e**, **g**) and subepimyocardial (**f**, **h**) circumferential strain values, strain curves, and color-M-Modes are shown. Line graphs of apical (blue) and basal rotation (magenta) as well as net rotation (twist) (white) (**i**) and corresponding line graphs of rotation rate (**j**) document normal findings

The importance of the described echocardiographic findings in SARS-CoV-2-infected patients is underlined by the comparison with data sets of age-matched controls (*n* = 20) showing normal findings of LV deformation in all healthy individuals (Table 3). Schemes of normal LV deformation graphs are illustrated in Fig. 5. Normal curves of rRS, rCS, and LV rotation of a representative healthy individual are shown in Fig. 7.

**Table 3:** Analyses of myocardial rotational LV deformation in a control group of healthy individuals (*n* = 20)

Parameter	LV segments (myocardial layers)	Mean values ± SD
GRS (%)	Apical LV segments	31.4 ± 7.1
Rotation (°)	Apical LV segments	5.9 ± 2.7
GCS (%)	Apical LV segments (subepimyocardial layers)	− 16.4 ± 4.8
GCS (%)	Apical LV segments (subendomyocardial layers)	− 36.2 ± 7.4
GRS (%)	Basal LV segments	30.0 ± 6.7
Rotation (°)	Basal LV segments	− 6.2 ± 2.8
GCS (%)	Basal LV segments (subepimyocardial layers)	− 15.3 ± 4.1
GCS (%)	Basal LV segments (subendomyocardial layers)	− 34.5 ± 5.4

Mean values of radial and circumferential strain as well as rotation of the apical and basal LV segments are shown: global radial strain of apical LV segments (GRS apical), apical rotation, global circumferential strain of apical LV segments (GCS apical), global radial strain of basal LV segments (GRS basal), basal rotation, and global circumferential strain of basal LV segments (GCS basal)

## Discussion

The main findings of the present study are:

1. Despite normal LVEF, most of SARS-CoV-2-infected patients showed abnormal LV deformation.
2. In patients with SARS-CoV-2 infection, especially basal LV segments seem to be affected. rRS, rCS, and rotation curves show abnormal patterns that may be described as “basal reverse tako-tsubo like-syndrome”.
3. Basal circumferential LV myocardial involvement was usually transmural.
4. Abnormal deformation patterns were also observed in SARS-CoV-2-infected patients with mild/moderate symptoms.

### Detection of acute myocarditis by echocardiography

In several case reports, speckle tracking is described as a useful tool to diagnose AM [22–25]. However, CMR is still the gold standard to diagnose AM according to Lake Louise criteria and T1-/T2-mapping [17, 18]. In AM induced by cardiotropic viruses, e.g., coxsackie, echo, influenza, Epstein–Barr, etc., myocardial damage is predominantly observed in the subepimyocardial layers, as documented by CMR and necropsy studies [17, 18, 26]. In AM induced by hyperinflammation or cytokine storm, transmural involvement can be observed [6]. In general, AM is characterized by a very high variability of myocardial involvement resulting in various patterns of myocardial deformation with respect to localization, myocardial transmurality, and/or AM severity [27–29]. Furthermore, different viral infections may affect different myocardial structures within the LV wall architecture probably inducing characteristic deformation patterns.

Longitudinal deformation is mainly described by subendomyocardial, circumferential deformation, and rotation mainly by subepimyocardial layers [29–31]. It has to be considered that inner LV layers do significantly more contribute to the overall contraction than outer layers. The problem of AM detection by echocardiographic LV deformation—especially at early stages—is the predominant myocardial involvement of the outer quarter subepimyocardium, which only contributes about 10% to the overall contraction. Thus, in suspected AM, normal LV systolic function will be observed if it will be analyzed by LVEF or longitudinal deformation, e.g. GLS.

Although RV GLS was mildly reduced (between − 17 and − 23%) in four patients with severe and two patients with mild/moderate symptoms in comparison to reported normal mean vales of RV GLS (28.5 ± 4.8), RV GLS was at least in line with the lowest expected value for patients < 50 years in all 18 patients with SARS-CoV-2-infection [32, 33]. It can be assumed that RV function might be earlier impaired in patients with AM due to the thinner RV wall. The reduction of RV function is known as a prognostic factor in SARS-CoV-2-infected patients [34, 35].

The analysis of LV deformation including circumferential strain and rotation may improve the echocardiographic diagnostic approach in AM [24, 27, 28]. However, the use of myocardial deformation is not yet widely implemented in patients with AM [36, 37]. Recently, the authors of the present study demonstrated the feasibility to define deformation patterns for radial and circumferential strain as well as rotation in a cohort of healthy volunteers and professional athletes [19]. As also performed in the present study, several prerequisites have to be considered during image acquisition and post-processing analyses to exclude artifacts; for example, the acquisition of standardized short-axis views perpendicular to LV long axis, the documentation of apical rotation at the apical third of the left ventricle, the adjustment of the tracking area to exclude paracardial structures, as well as parts of the mitral valve [37]. Furthermore, the results of rotational deformation need to be counterchecked by multiple documentations to differentiate artifacts from deformation abnormalities as sequelae of SARS-CoV-2-induced myocardial involvement. In the future, 3D voxel tracking might also represent a new option to analyze LV rotation in patients with insufficient parasternal acoustic window. However, the limited spatial resolution of 3D echocardiography is still challenging.

### Is there a specific deformation pattern in SARS-CoV-2-induced myocarditis?

The present study suggests that SARS-CoV-2-induced AM may be detected by deformation abnormalities using speckle tracking echocardiography. SARS-CoV-2-induced myocardial involvement often shows specific LV deformation patterns due to pronounced edema and/or myocardial damage in basal LV segments. Normal global longitudinal deformation and abnormal basal rLS underline the assumption that the early stage of SARS-CoV-2-induced myocarditis is characterized by a subepimyocardial involvement. Furthermore, abnormalities of basal rRS and rCS suggest a primarily basal subepimyocardial involvement. Transmural myocardial involvement obtained by circumferential strain analysis may indicate an advanced stage of the disease at the time of investigation. Comparable to Fabry and Friedreich disease [38, 39], SARS-CoV-2- induced myocardial involvement was predominantly observed in the basal/mid infero-/anterolateral LV segments, which could be partially explained by a pronounced—presumably hydrostatic—edema formation due to the supine position of the patient. The finding of a “reverse basal tako-tsubo-like syndrome” of basal LV segments might also be explained by the edema, which leads to abnormal basal rRS curves without any alterations during systole [15]. Comparing SARS-CoV-2-induced LV deformation patterns with the corresponding CMR findings in the present study, different LV deformation patterns are observed in comparison to AM patients caused by other cardiotropic viruses [17, 18, 40].

Interestingly, abnormal LV deformation patterns were still observed in all three patients after recovery from the acute stage indicating residual myocardial involvement. Furthermore, CMR showed late enhancement predominantly in the basal inferolateral/anterolateral LV segments indicating myocardial fibrosis or scars, respectively. Myocardial fibrosis might serve as potential arrhythmogenic substrates in long-term follow-up of SARS-CoV-2-induced AM patients. In consequence, CMR follow-up may be considered for patients after SARS-CoV-2-infection, who still show at least two major criteria of LV deformation abnormalities to assess residual scar formation or myocardial fibrosis.

The observations of pathological mid basal rotational LV deformation patterns in a high percentage of SARS-CoV-2-infected patients seem to be important, but are obviously not a proof of a specific myocardial involvement in these patients.

The main limitation of the present study is the relatively small number of patients. 3D echocardiography could not routinely be performed at the isolation wards. Due to the evolving situation, the data of follow-up and CMR are limited and should be considered hypothesis generating. The strengths of the present study are the new findings of myocardial involvement due to abnormalities of LV deformation obtained by modern echocardiography in patients with SARS-CoV-2. However, the data about patients with SARS-CoV-2-infection should be interpreted carefully, because differences between percentage observations within the respective (sub)groups cannot show statistical significance.

## Conclusions

Myocardial involvement due to SARS-CoV-2-induced AM might be characterized by specific LV deformation patterns—even in patient with mild/moderate symptoms. The detection of myocardial involvement by speckle tracking echocardiography is feasible at the acute stage of COVID-19 which may improve the early detection of myocardial involvement, risk stratification, and potentially cardio-protective treatment. Speckle tracking echocardiography might also be suitable to detect residual myocardial involvement after acute stage of SARS-CoV-2-infection. The present study sets the stage for follow-up studies to determine the prognostic value of these alterations in patients with SARS-CoV-2 infection.
